# Data-Driven Math Model of FLT3-ITD Acute Myeloid Leukemia Reveals Potential Therapeutic Targets

**DOI:** 10.3390/jpm11030193

**Published:** 2021-03-11

**Authors:** David J. Wooten, Melat Gebru, Hong-Gang Wang, Réka Albert

**Affiliations:** 1 Department of Physics, Pennsylvania State University, University Park, PA 16802, USA; dzw347@psu.edu; 2 Department of Pediatrics, Penn State College of Medicine, Hershey, PA 17033, USA; mtg194@psu.edu (M.G.); hwang3@pennstatehealth.psu.edu (H.-G.W.)

**Keywords:** acute myeloid leukemia, Boolean model, drug resistance, network

## Abstract

*FLT3*-mutant acute myeloid leukemia (AML) is an aggressive form of leukemia with poor prognosis. Treatment with *FLT3* inhibitors frequently produces a clinical response, but the disease nevertheless often recurs. Recent studies have revealed system-wide gene expression changes in *FLT3*-mutant AML cell lines in response to drug treatment. Here we sought a systems-level understanding of how these cells mediate these drug-induced changes. Using RNAseq data from AML cells with an internal tandem duplication *FLT3* mutation (*FLT3*-ITD) under six drug treatment conditions including quizartinib and dexamethasone, we identified seven distinct gene programs representing diverse biological processes involved in AML drug-induced changes. Based on the literature knowledge about genes from these modules, along with public gene regulatory network databases, we constructed a network of *FLT3*-ITD AML. Applying the BooleaBayes algorithm to this network and the RNAseq data, we created a probabilistic, data-driven dynamical model of acquired resistance to these drugs. Analysis of this model reveals several interventions that may disrupt targeted parts of the system-wide drug response. We anticipate co-targeting these points may result in synergistic treatments that can overcome resistance and prevent eventual recurrence.

## 1. Introduction

Acute myeloid leukemia (AML), characterized by the pathological accumulation of myeloblast cells in blood or bone marrow, is a heterogeneous and aggressive form of leukemia. About 30% of AML cases carry a mutation in the *FLT3* gene, which encodes a receptor critical for normal hematopoiesis [1]. By far the most common mutation is an internal tandem duplication (*FLT3*-ITD), which occurs in about 25% of all AML cases [1], a mutation placing patients in a poor prognosis category [2]. Highly specific *FLT3* inhibitors are therapeutically promising [1,2], though the disease often recurs.

Recent experimental results have suggested that while *FLT3*-inhibition can kill *FLT3*-ITD cells, some cells survive and become drug tolerant persisters (DTPs) [3,4]. Targeting the therapeutic vulnerabilities of drug-tolerant FLT3 mutant AML cells can enhance the anti-leukemic efficacy of FLT3 inhibitors to eliminate minimal residual disease, mutational drug resistance and relapse. The mechanisms underlying this phenotypic change are not fully understood. A recent study found that DTPs exhibit the upregulation of inflammation pathways, and combination treatment with quizartinib (a *FLT3* inhibitor) and dexamethasone (a glucocorticoid that reduces inflammation) was synergistic [4]. This is an example of reprogramming therapy, in which the phenotypes or gene expression patterns induced by one drug are countered by another simultaneous intervention.

The idea of reprogramming cancer cells into drug-sensitive states [5,6,7,8,9] or even non-malignant states [10,11] has become increasingly promising. Reprogramming drug-sensitivity follows from the hypothesis that drug treatment induces reversible, system-wide gene expression and epigenetic changes, causing cells to achieve a resistant or tolerant subtype [12,13]. Targeting these changes and reverting them may then reprogram the cells. With this view, we seek to gain a systems-level understanding of the gene expression and phenotypic changes of *FLT3*-ITD AML cells in response to drug treatment with quizartinib and dexamethasone, and their evolution into DTPs.

To this end, we identified several modules of co-expressed genes that correspond to different treatment conditions with quizartinib, dexamethasone, or their combination. Based on genes within these modules, we built a network model of *FLT3*-ITD AML drug response. Using data-driven tools, we derived a probabilistic, dynamical gene regulatory model that recapitulates the expression changes of AML cells following these drug treatments and can be used to predict the effects of perturbations and interventions in the cells. We focused on identifying interventions that downregulate modules associated with drug resistance, and upregulate modules associated with cell death. The interventions we identified represent promising strategies to improve response to *FLT3* inhibitors in *FLT3*-ITD AML.

## 2. Materials and Methods

### 2.1. Data Acquisition and Processing

RNAseq data of MV4-11 cells were collected by M. Gebru, as described in [4], and previously made publicly available on GEO (GSE116432). Data consisted of triplicate measurements, each of (1) 10 nM quizartinib treatment for 48 h, (2) 10 nM quizartinib treatment for five days, (3) 100 nM dexamethasone treatment for 48 h, (4) combination 10 nM quizartinib + 100 nM dexamethasone for 48 h (we refer to this combination as Quiz + Dex), (5) Quiz + Dex for five days (quizartinib for five days and dexamethasone added on day 3 because the combination for 5 days would kill almost all cells), and (6) DMSO (GEO: GSE116432). Data were transformed as log(1 + FPKM). Only transcripts with a matched HGNC symbol were kept.

### 2.2. Weighted Gene Co-Expression Network Analysis

**Figure 1:**
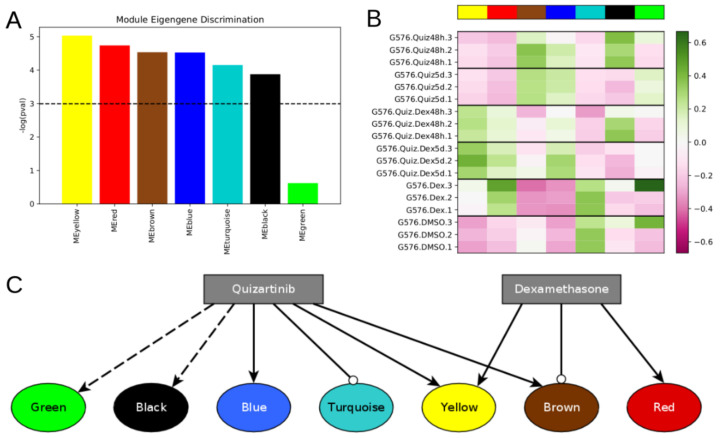
Differentially expressed modules respond differently to different treatment conditions. (**A**) WGCNA identified seven gene co-expression modules from the DMSO and drug treated *FLT3*-ITD AML expression dataset, six of which are differentially expressed across the six different treatment conditions. (**B**) Heatmap showing module eigengene expression for each module in each sample. High module eigengene expression reflects high average expression of genes within that module. (**C**) Qualitative model showing the effect of each drug on the expression of genes within each module. Arrow-tipped edges indicate activation, while circle-tipped edges indicate repression. The dotted edge from quizartinib to the black module reflects the observation that black module genes are upregulated at 48 h by quizartinib, but become downregulated again by five days of treatment. The dotted edge from quizartinib to the green module reflects that the green module is not upregulated after 48 h, but is after 5 days.

We used v1.69 of the WGCNA package in R v4.0.2. We used the pckSoftThresold function with a “signed” network type to identify power = 10 as the smallest power that achieved a scale-free R^2^ value >= 0.9 (Figure S1). We built a topological overlap matrix using a signed adjacency matrix obtained from power = 10. Genes were hierarchically clustered using the “average” method, and genes were assigned to co-expression modules using WGCNA’s cutreeDynamic function with deepSplit = 2, pamRespectsDendro = FALSE, and minClusterSize = 100. This analysis resulted in seven modules of co-expressed genes (Figure 1A and Figure S1). Following WGCNA convention, the modules are denoted by color: turquoise (7219 genes), blue, yellow, brown, green, black (164 genes).

### 2.3. Molecular Biology of the Cell (MBCO) Ontology Analysis

MBCO analysis was completed using the source code from https://github.com/SBCNY/Molecular-Biology-of-the-Cell/commit/9ff6c87 (accessed on 15 March 2020). The background gene set consisted of all genes from the RNAseq dataset, and ontology analysis was performed independently for each WGCNA gene module. Enrichment results are given in Figures S2–S8 and File S2.

### 2.4. Network Construction

To build the network, we integrated interactions from multiple databases that aggregated literature-based or predicted interactions, SIGNOR [14], TRRUST [15], and RegNetwork [16], as well as published networks related to AML [14,17], NFKappaB signaling [17], NOTCH signaling [18], tumor promoting inflammation [19,20], and apoptosis [20].

Many of these network resources have minor variations in gene names or use different aliases for different genes. We applied two methods to transform gene names from different sources into a common space so that all interactions with a given gene may be identified, even if the different sources use different names for that gene. First, we considered that many sources use different capitalization, or interchangeably use “.”, “-”, or “_” characters. To address this, we capitalized all characters in each gene name, and removed all “.”, “-”, and “_” characters. Second, to match gene aliases across different network sources, we used three separate gene name alias data sources, including Entrez Homo_sapiens gene info (https://ftp.ncbi.nih.gov/gene/DATA/GENE_INFO/Mammalia/Homo_sapiens.gene_info.gz (accessed on 25 October 2020)), BioMart from Ensemble (https://useast.ensembl.org/biomart (accessed on 25 October 2020)), and HGNC (https://www.genenames.org/ (accessed on 25 October 2020)). Each source includes multiple aliases for each gene name. We constructed a gene name alias graph whose nodes represent gene names, and in which each edge represents that two nodes are aliases for the same gene from one of those resources. Within this alias graph, if there exists a path from one node to another, it indicates they refer to the same gene.

There were several properties we wanted the final AML network topology to have, and the strategy we used to build the network was refined until we reached a network that satisfied these properties. First, we wanted the network to be large enough to capture enough regulatory details (e.g., more than about four nodes per module, or about 30 nodes total), but not too large to be able to model or simulate well (e.g., fewer than about 200 nodes). Second, we wanted all seven gene co-expression modules to be similarly represented, even though some modules are much larger than others (turquoise and blue have thousands of genes each but green and black only have a few hundred genes each). Third, we wanted the in-degree of nodes to not be too large (e.g., more than about 7 incoming edges). This is because a Boolean regulatory function with N inputs has 2^N^ possible input conditions for which an output value must be specified. When inferring Boolean functions of 7 or more variables using the BooleaBayes algorithm, the probability that any given sample constrains a given input condition becomes extremely small, and the resulting Boolean function becomes nearly completely stochastic.

The process we used to build the final network is shown in Figure S9. First, we merged all the network sources (e.g., SIGNOR) into a single large network, wherein nodes that were aliases of one another from different sources were merged into a single node. This network contained 8614 nodes and 35,710 edges. Of the nodes, 2374 had non-zero out-degrees and represented a gene from the RNAseq dataset. We then extracted subgraphs consisting of only genes from the brown, red, green, yellow, and black modules. We focused on these first as they are smaller modules than blue and turquoise, and we wanted to include as many of these nodes as possible to ensure they are well represented in the final network. We merged these five subgraphs together, which resulted in a disconnected graph. This graph contained only two components with five or more nodes, one of which consisted of 18 green nodes, the other consisted of 53 brown nodes. We hypothesized that nodes from the red, yellow, or black modules may be connected into these components through paths (successions of edges) containing nodes not in the brown, red, green, yellow, or black modules. For example, no blue or turquoise nodes had been included at this point. We searched for paths of no more than four nodes that could connect nodes from the red, yellow, or black modules into the above-mentioned components (Figure S9B). Anytime multiple paths were found, we only added the shortest path. If there were multiple equally short paths, all were added.

We removed all sink nodes because they do not feed back into the dynamics of the network, and thus cannot be drivers. The resulting graph contained 186 nodes and 888 edges. Of the nodes, 52 belonged to the brown module, 34 to turquoise, 23 blue, 21 yellow, 20 red, 15 green, and 9 black, while the others belonged to no module. This satisfied our goals of having approximately equal representation of the different modules, and not too few nor too many nodes. However, many nodes in the resulting graph had extremely high in-degree. For example, RELA had 43 in-edges, TP53 had 37, and FOXO3 had 36. The Boolean regulatory update function for RELA would then have 2^43^ ~= 10^12^ conditions that must be specified, which would be impractical, and impossible given available data.

To avoid such excessively high in-degree nodes in the network, we calculated an edge score that we used to retain only the most confident edges. We set a threshold that must be exceeded to include an edge, and made this threshold increase as more in-edges are added to a node. This process preserves a node’s regulators if it has low in-degree, but provides an increasingly strict criterion for edges to be included as the in-degree becomes larger.

The edge score was based on the following factors: (1) whether or not the source node is a transcription factor (TF), (2) the number of references supporting the edge, (3) the number of different databases (e.g., SIGNOR) or literature-based networks that included the edge, and (4) the edge confidence given by the network resource, including “belief” (networks from Indra) or “score” (SIGNOR, TRUUST). Regarding point (1), if the source node is a TF, the edge score is multiplied by $TF_{MUL}=2$. If not, $TF_{MUL}=1$. Regarding point (4), for network resources that did not provide edge confidence, the confidence was assumed to be 0. With these metrics, the edge score was calculated as:

$$score=\left(N_{references}+N_{resources}+10\cdot \frac{confidence}{N_{edges}}\right)\cdot TF_{MUL}$$

The minimum possible score was 2, as $N_{references}$ and $N_{resources}$ were at least each 1 for every edge. For each node, all its incoming edges were scored and ordered from highest to lowest. The (up to) three edges with the three highest scores were always included. Following these, each subsequent edge was included if $score>N_{IN-EDGES}-1$. For example, given five incoming edges with scores (5, 4.5, 4, 3, 2), the first three edges (scores 5, 4.5, and 3) are automatically included. The next edge has score = 3, which is greater than $N_{IN\;EDGES}-1=3-1=2$, so it is included. The next edge has score = 2, which is no longer greater than $N_{IN\;EDGES}-1=4-1=3$, so it, and any lower score edges, would not be included.

Finally, once again, all sink nodes, or nodes that do not belong to a component of at least size = 4, were removed. The final network had 106 nodes and 270 edges.

### 2.5. Regulatory Function Inference using the BooleaBayes Algorithm

Using the transcription data from the RNAseq dataset, the node activation data constructed as described in the next section, and the network topology, we inferred probabilistic Boolean regulatory functions using the BooleaBayes algorithm as described in [6]. Briefly, BooleaBayes tries to find Boolean logic functions consistent with steady-state gene expression data and a network topology. As BooleaBayes needs normalized expression, RNAseq data for each gene were normalized between 0 and 1 by setting all values less than the 20th percentile to 0, all values above the 80% to 1, and all values in-between were linearly interpolated between 0 and 1.

BooleaBayes infers a probabilistic Boolean regulatory function for each node in the network. For each function, all input regulators are assigned a significance value by BooleaBayes, defined as the maximum possible (absolute value) difference in output value the regulator can make if it switches from OFF to ON. We set a minimum threshold of 0.1 for this value. With this threshold, each regulator must, in at least one condition, mean the difference between a 0.45 or less output, and a 0.55 or greater output.

When fitting the function for a node, if at least one regulator did not exceed this threshold, the regulator with the lowest significance was removed, and the function was inferred again using only the remaining regulators. This process was repeated until either all regulators exceeded the minimum significance threshold, or no regulators remained. In the latter case, the target node becomes a source node for later analyses.

### 2.6. Extension of BooleaBayes to Post-Translational Regulation

Unlike previous work with BooleaBayes, which focused purely on transcriptional regulation, the AML network includes post-translational modifications. However, the expression data only include transcription quantification. To apply the BooleaBayes algorithm, we must separate the probability of a node being transcribed from the probability of a node being active. For instance, if node A regulates node B, node A may be transcribed but not active, in which case the input value of node A into node B’s Boolean function should be OFF.

To this end, we first distinguished for each edge whether it represented transcriptional regulation or post-translational regulation. An edge whose source node is a transcription factor according to [21] was considered to be a transcriptional edge. All other edges were considered as post-translational. Post-translational edges were assigned as positive (activating) or negative (de-activating) based on edge annotations from the source network. For example, SIGNOR and Indra edges indicate whether the regulator up-regulates or down-regulates the target. For edges with no consistent database annotation, edge weights were obtained from literature search when possible, or assumed to be positive if no specific supporting information could be found.

Any node that is only transcriptionally regulated is assumed to be active as long as it is transcribed. All nodes that are post-translationally regulated (such as a node named “X”) were split into transcript (X_T) and active protein (X_A) forms. Any outgoing edges (regulatory effects) from nodes that have _T and _A forms are assumed to come only from the _A form.

To fit BooleaBayes functions, the values from the RNAseq data are used directly for X_T. Values for X_A for each sample must be determined prior to applying BooleaBayes, so that the target nodes of X use X_A for their training data, instead of X_T. We assumed that protein post-translational activation follows an inhibitory dominant form. For example, if X_A is activated by nodes J and K, and deactivated by node M, we say

X_A = X_T and (J or K) and not M

(or J_A, K_A, or M_A, if any of those regulators require an activated form). As X_T, J, K, and M are not strictly Boolean variables, but rather probabilities, we transform this into a sloppy logic form by replacing “or” with “+”, “and” with “*”, and “not” with “1-”. Further, each term is strictly held within 0 and 1. Thus

X_A = X_T * min(J+K, 1) * max(1-M, 0)

More generally, as long as X has at least one activator we say

X_A = X_T * min(sum(ACTIVATORS_X), 1) * max(1-sum(INHIBITORS_X), 0)

while, if X has no activators, we say

X_A = X_T * max(1-sum(INHIBITORS_X), 0)

This distinction prevents nodes that have no activators from always being inactive—they are assumed to be active unless deactivated. We constructed such an equation for every node that must be activated. These equations formed a system of nonlinear algebraic equations which we solved numerically using the scipy.optimize.fsolve() function in Python v3.8, with an initial guess for each node of X_A = X_T. The resulting values of X_A were then added to the gene expression dataset to be used for inferring BooleaBayes functions for any node regulated by an _A form of a regulator.

### 2.7. Identification of Pseudo-Attractors

Pseudo-attractors of a probabilistic discrete system are states, or collections of states, that the system keeps revisiting. Expressed more technically, pseudo-attractors are collections of states for which transitions into them are more likely than transitions out, along every axis. The sum of forward and backward transition probabilities between two BooleaBayes states always adds up to 1. Therefore, if a transition is more likely into a state than out of a state, the out-transition will be less than 0.5. Thus, pseudo-attractors of a BooleaBayes-inferred system will correspond to the attracting strongly connected components of the state transition system, for which all transitions with probability less than 0.5 are removed. This corresponds exactly to the attractors of the closest approximating deterministic Boolean system, obtained by rounding all probabilities to the nearest 0 or 1—all transitions in the probabilistic system with probability less than 0.5 are absent.

Thus, to identify pseudo-attractors of the probabilistic AML drug network, we approximated each BooleaBayes-inferred update function to its closest deterministic function. We used the AttractorRepertoire module from the StableMotifs [22] python package to find attractors of the deterministic system. The system has a very large number of source nodes (nodes with no regulators), which allows many attractors. To isolate the attractors most relevant to AML drug response, we determined the Boolean state of these source nodes for each of the six experimental conditions by averaging their probability to be ON or OFF from the data. For each node, if it was more likely to be ON across the three replicates, we plugged in the value ON to the deterministic system and propagated its value through the Boolean update functions, and likewise for OFF.

### 2.8. Node Interventions

We sought to understand how interventions that target specific nodes influence the stability of WGCNA gene modules. We considered two types of interventions: holding a node in the OFF (0) state, akin to knockout (KO) and holding a node in the ON (1) state, akin to constitutive activation (CA). We assumed that any intervention targeting a gene that was separated into transcribed and active protein forms applied to both forms. During simulations, the states of controlled nodes were held constant, and other nodes were updated as in the WT system.

### 2.9. Definition and Calculation of Influence Index

Systematic in silico intervention experiments require a significant number of computational resources, thus we wanted to prioritize the most likely candidates for up- or down-regulating a target module. To this end, we calculated an “influence index” for each node-intervention-module tuple, for example the tuple “GSK3B, KO, blue module”. The influence index is designed to estimate how likely it is that the influence of a node-intervention on the node’s direct targets aligns with the up- or down-regulation goal of a specified module.

The influence index is based on the concepts of necessary and sufficient regulation. If node A = ON is necessary for node B = ON, this means that A = OFF implies B = OFF. Conversely, if node A = ON is sufficient for node B = ON, this means that A = ON implies B = ON [23]. For each edge we developed scores quantifying the likelihood that the edge represents necessary regulation or sufficient regulation. In total we calculated four scores for each edge: (1) the source is necessary for the target to be ON (called $N_{ON}$), (2) the source is sufficient for the target to be ON (called $S_{ON}$), (3) the source is necessary for the target to be OFF (called $N_{OFF}$), and (4) the source is sufficient for the target to be OFF (called $S_{OFF}$). These scores are based on the average value of the probabilistic function output when the node at the source of the edge is ON ($avg_{ON}$), or the source node is OFF ($avg_{OFF}$). For example, consider a node C whose regulatory function is $f\left(A,B\right)$. For the edge A→B, $avg_{ON}=\frac{f\left(1,0\right)+f\left(1,1\right)}{2}$ while $avg_{ON}=\frac{f\left(0,0\right)+f\left(0,1\right)}{2}$.

Using this definition of $avg_{ON}$ and $avg_{OFF}$, $N_{ON}$, $S_{ON}$, $N_{OFF}$, and $S_{OFF}$ were calculated as follows:

If an edge represents overall positive regulation (meaning that switching the source node from OFF to ON increases the likelihood that the target turns on)

$$\begin{array}{l} N_{ON}=1-avg_{OFF}\\ S_{ON}=avg_{ON}\\ N_{OFF}=0\\ S_{OFF}=0 \end{array}$$

Conversely, if an edge represents overall negative regulation (meaning that switching the source node from OFF to ON decreases the likelihood that the target turns on)

$$\begin{array}{c} N_{ON}=0\\ S_{ON}=0\\ N_{OFF}=avg_{OFF}\\ S_{OFF}=1-avg_{ON} \end{array}$$

To illustrate these definitions, consider a node D with deterministic Boolean update function f(A,B,C) = A or (B and C). This function means that node D will turn on if A is ON or if B and C are simultaneously ON. For the edge A → D, we can calculate

$$\begin{array}{l} avg_{ON}=\frac{f\left(1,0,0\right)+f\left(1,0,0\right)+f\left(1,1,0\right)+f\left(1,1,1\right)}{4}=1\\ avg_{OFF}=\;\frac{f\left(0,0,0\right)+f\left(0,0,0\right)+f\left(0,1,0\right)+f\left(0,1,1\right)}{4}=0.25 \end{array}$$

A is a positive regulator of D, so $N_{ON}=1-0.25=0.75$, $S_{ON}=1$, and $N_{OFF}=S_{OFF}=0$. This means that in 75% of input conditions A would be necessary to turn D ON (only when B=C=1 does D turn ON without A). Conversely, A is sufficient to turn D ON in all input conditions. Finally, A is never sufficient nor necessary to turn D OFF, as A is a positive regulator of D.

With this, we define the influence index of each node-intervention-module tuple using one of the following formulas:

Source node intervention: KO; Target module goal: DOWN

$$InfluenceIndex=\;\sum \left[\left(N_{ON}-N_{OFF}\right)+0.5\cdot \left(S_{ON}-S_{OFF}\right)\right]$$

Source node intervention: KO; Target module goal: UP

$$InfluenceIndex=\;\sum \left[\left(N_{OFF}-N_{ON}\right)+0.5\cdot \left(S_{OFF}-S_{ON}\right)\right]$$

Source node intervention: CA; Target module goal: DOWN

$$InfluenceIndex=\;\sum \left[\left(S_{OFF}-S_{ON}\right)+0.5\cdot \left(N_{OFF}-N_{ON}\right)\right]$$

Source node intervention: CA; Target module goal: UP

$$InfluenceIndex=\;\sum \left[\left(S_{ON}-S_{OFF}\right)+0.5\cdot \left(N_{ON}-N_{OFF}\right)\right]$$

where in each case the sum is over all target nodes of the perturbed node that are in the target gene module. The higher weight on necessary edges in KO interventions reflects the fact that turning OFF a necessary regulator is sufficient to control its output. The higher weight on sufficient edges in CA interventions reflects the fact that turning ON a sufficient regulator is sufficient to control its output.

### 2.10. Analyzing the Effect of Node Interventions

In contrast to attractors of deterministic systems, our stochastic model can evolve away from pseudo-attractors (i.e., pseudo-attractors are not trap spaces). We start simulations from a system state that corresponds to the average state of all pseudo-attractors associated to a given experimental condition, and examine how many steps are required for a given module’s overall expression to increase or decrease relative to its start state.

To accomplish this, we quantify a module’s “activation” as the fraction of nodes in the module that are ON. For the purpose of this calculation, we exclude all source nodes, as those nodes cannot be activated or silenced based on interventions of other nodes, and are, therefore, insensitive to any perturbation. During simulations we very rarely observed a module achieve more than 3/4 of non-source nodes becoming ON. We thus considered switches between states that have low module activation (fewer than 1/4 non-source nodes are ON) and intermediate module activation (between 1/4 and 3/4 non-source nodes are ON).

We simulated the dynamics of the WT system by starting from a pseudo-attractor and updating a single, randomly selected node at each time step [6]. For modules that start in the low activation state, we counted how many steps were required for the module to switch to the intermediate state for the first time. For modules that start in the intermediate state, we instead counted how many steps were required to switch to the low activation state for the first time. We repeated these simulations, restarting from the start state, 100 times. For each simulation, we updated the system 5000 times. If a module did not switch within that time, we assigned a value 5001. For subsequent statistical analyses, we used a non-parametric ordinal test, so in most cases it does not practically matter how much above 5001 it really would have been.

We then chose a set of interventions to test, based on analysis of the network and influence index of various nodes. We considered single node KO or CA, or combinations of multiple nodes individually controlled. As in the WT system, we performed 100 iterations of 5000 steps, counting how many steps were required for a module to switch for the first time. We used a two-sided Mann–Whitney U test to test whether the average number of steps from the intervention simulations was statistically different from the WT. All *p*-values were FDR-corrected using the Benjamini–Hochberg (BH) method, and the threshold for significance was defined as BH-adjusted *p* < 0.05.

Following intervention, if a module requires more steps before it switches from low to intermediate activation, or vice versa, compared to WT, then the module’s original state was stabilized by the intervention. If the module began in the low state, we then classified the intervention as down-regulating. If the module began in the high state, we classified the intervention as up-regulating. Conversely, if an intervention makes a module require fewer steps to switch, then the module’s original state was destabilized by the intervention. If the module began in the intermediate state, we classified the intervention as down-regulating. If the module began in the low state, we classified the intervention as upregulating.

## 3. Results

### 3.1. Identification of Gene Co-Expression Modules Associated with Distinct Treatments

We analyzed an RNAseq dataset [4] consisting of MV4-11 cells (a *FLT3*-ITD AML cell line) exposed to six different treatment conditions. These included triplicate measurements each of (1) 10 nM quizartinib treatment for 48 h, (2) 10 nM quizartinib treatment for five days, (3) 100 nM dexamethasone treatment for 48 h, (4) combination 10 nM quizartinib + 100 nM dexamethasone for 48 h (we refer to this combination as Quiz + Dex), (5) Quiz+Dex for five days (quizartinib for five days and dexamethasone added on day 3), and (6) DMSO (GEO: GSE116432). Previous work found that dexamethasone and quizartinib in combination were synergistic in *FLT3*-ITD cells [4].

Applying weighted gene co-expression network analysis (WGCNA) [24] to this gene expression dataset, we identified seven modules of co-expressed genes (Figure 1A and Figure S1). WGCNA assigns color names to each module. The modules we identified ranged in size from 164 genes (black module) up to 7219 genes (turquoise module). The genes in each module are given in File S1.

Given a WGCNA gene module, the module’s eigengene (defined as the first principal component) is commonly used as a single metric capturing the overall expression of all genes within that module. Based on module eigengene expression, we found that six modules were statistically differentially expressed across treatment conditions (Figure 1A, Kruskal–Wallis test, BH-adjusted *p*-value < 0.05): the yellow, red, brown, blue, turquoise, and black modules.

Of these modules, we find that the yellow module is upregulated (relative to DMSO) by all treatments, most significantly by the combination of dexamethasone + quizartinib (Figure 1B). The red module is upregulated by dexamethasone, with or without the addition of quizartinib, while we detected no response of this module to quizartinib alone. The blue module is upregulated by quizartinib, with or without the addition of dexamethasone, while we detected no response of this module to dexamethasone alone. Opposite to blue, the turquoise module is downregulated by all treatments, including quizartinib. Finally, the black module is upregulated after 48 h of treatment with quizartinib (with or without dexamethasone) but returns to DMSO levels after 5 days of treatment. Though the green module was not significantly differentially expressed, we noticed that, within the triplicate measures in both DMSO and dexamethasone treatment, one sample of each appears to be a clear outlier in the green module eigengene expression (Figure 1B). Without those samples, the green module is upregulated following quizartinib treatment for 5 days. Thus, the green module may still be relevant to understanding AML drug response, and we consider its possible role later. These results are summarized as interactions between the drugs and modules in Figure 1C, which shows that dexamethasone reverses quizartinib-induced upregulation of the brown module but does not reverse other modules that are affected by quizartinib.

### 3.2. Ontology Analysis Reveals Biological Processes Unique to Each Module

To uncover the biological character of each gene module, we performed ontology enrichment analysis using the Molecular Biology of the Cell Ontology (MBCO) method [25]. This analysis searches not only for enriched sub-cellular processes, but enriched relationships between processes. The results of MBCO analysis are reported in Figures S2–S8, and in File S2. These analyses revealed several biological processes and pathways that become activated by different drug treatments, that may play critical roles in mediating AML drug response.

We found the yellow module was enriched for cell–cell communication, especially via NOTCH signaling, as well as extracellular matrix homeostasis. The red module was strongly enriched for extracellular matrix homeostasis, including collagen biosynthesis and crosslinking. The brown module was enriched for immune response activation and actin and lamellipodium structure. Collectively, these three modules thus may be responsible for mediating the tumor microenvironment response through cell–cell communication, structural changes, and immune activation.

The blue module, which is upregulated by quizartinib, was highly enriched in drug export and cellular detoxification, indicating a potentially essential role in mediating cell survival following treatment with quizartinib. The turquoise module was highly enriched in cell-cycle progression, suggesting a possible role in mediating proliferation. Treatment with quizartinib downregulates the turquoise module, which is consistent with the prior hypothesis that DTPs slow down their growth in the presence of drug [3,26,27]. Quizartinib’s simultaneous activation of the blue module, and downregulation of the turquoise module may be able to quickly lead to the emergence of DTPs, allowing cells to then acquire other changes, or change their environment, leading to more favorable cancer-cell survival. Dexamethasone treatment did not reverse the effect of quizartinib on these DTP-associated modules (Figure 1C).

The black module was found to be enriched in transcription and translation. The black module is upregulated by quizartinib in the short term, but returns to untreated levels by 5 days of treatment. This suggests that, early on in treatment, cells may quickly activate several gene transcription programs, but the activation of new programs may relax by day five as cells reach a new equilibrium.

Finally, though it was not significantly differentially expressed across subtypes, the green module was highly enriched in the regulation of apoptosis. As noted above, excluding two outlier samples, the green module is upregulated following quizartinib treatment for 5 days.

### 3.3. Network Analysis

#### 3.3.1. Construction of Gene Regulatory Network Governing AML Drug Response

**Figure 2:**
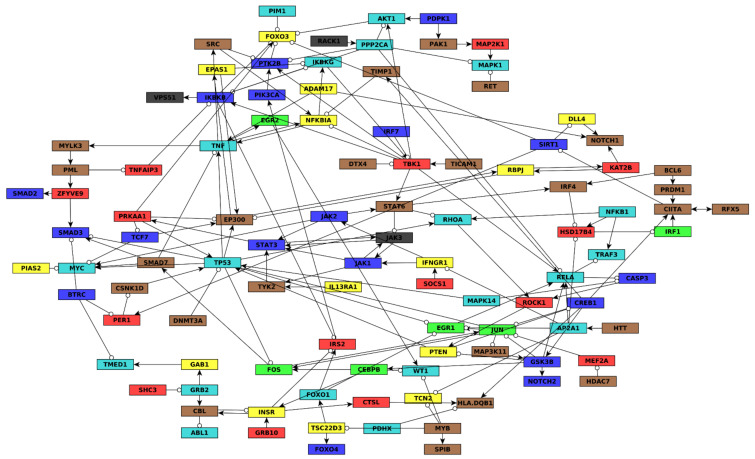
Gene regulatory network of *FLT*-ITD AML quizartinib and dexamethasone response. Nodes are genes and are colored by the WGCNA module to which they belong. Edges ending in arrows represent net positive regulation, while edges ending in circles indicate net negative regulation. Edge regulation sign is determined using the BooleaBayes algorithm (see Methods Section 2.5 and Section 2.6). The nodes in this network combine the transcript and the protein encoded by the same gene. For example, the node JUN has transcriptional regulators (FOS, CREB1, and MEF2A) and a posttranslational regulator (GSK3B). For nodes like this with post-translational modifications, the full network, shown in Figure S10, has separate nodes corresponding to their transcript and active protein.

To understand how cells mediate these drug-induced gene expression changes, we constructed a gene regulatory network model of highly differentially expressed genes from within each module. The full details of network construction are presented in Methods Section 2.4, Section 2.5 and Section 2.6 and an overview is in Figure S9. Briefly, we aggregated interactions from the public databases SIGNOR [14], TRRUST [15], and RegNetwork [16]. Based on the ontology analysis that implicated cell–cell communication, inflammation, and apoptosis, we also integrated published networks related to AML [14,17], NFKappaB signaling [17], NOTCH signaling [18], tumor promoting inflammation [19,20], and apoptosis [20]. The final network (Figure 2 and Figure S10) was constructed with the aim of avoiding over- or under-representation of any single module.

#### 3.3.2. Inference of Predictive Dynamic AML Drug Resistance Network Model and Drug-Induced Pseudo-Attractors

**Figure 3:**
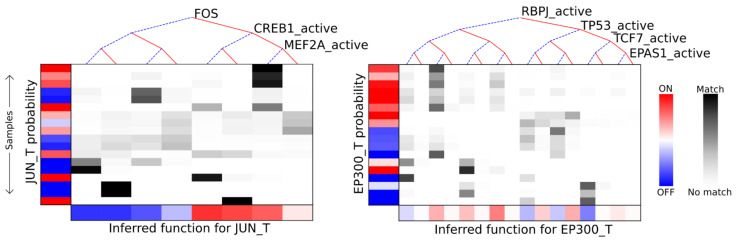
Examples demonstrating BooleaBayes regulatory function inference. Left: the inferred function for JUN transcription. JUN_T has three regulators: FOS, CREB1_active, and MEF2A_active. A Boolean function of 3 regulators has 2^3^ = 8 possible input configurations (e.g., FOS = 0, CREB1_active = 0, MEF2A_active = 0). Each column of the figure corresponds to one of the possible regulator configurations, from all regulators being OFF (left-most column) to all regulators being ON (rightmost column). Each row corresponds to one of the 18 AML samples. The red and blue colors along the far left show whether JUN_T (the target node) is ON or OFF in each sample. The white-black color scale shows how closely each sample (row) corresponds to a given input configuration (column). For example, the top three samples (rows) are most likely to correspond to FOS = 1, CREB1_active = 1, MEF2A_active = 0, as is shown by the black and dark grey cells in the first three rows of that column. In both samples, JUN_T is likely to be OFF (indicated by the blue color). Thus, the inferred regulatory function for JUN_T (bottom row) says that if FOS = CREB1_active = MEF2A_active = 0, JUN_T is very likely to turn OFF. Right: inferred function for EP300 transcription. Unlike JUN_T, there are many conditions for which there was no observed data, such as RBPJ_active = 0, TP53_active = 0, TCF7_active = 0, and EPAS1_active = 1 (second condition from the left). In these cases, the inferred rule has a near 50% chance for EP300_T to turn ON or OFF, as there are no data indicating what should happen in these cases.

We next sought to understand how the genes in the AML drug response network interact. To this end, we applied the BooleaBayes algorithm [6] to infer probabilistic regulatory functions for each node in the network (File S3). Briefly, BooleaBayes tries to find Boolean logic functions consistent with steady-state gene expression data and a network topology. In our case, the gene expression data are derived from normalization of the 18 AML RNAseq samples (see Methods Section 2.5) and the network topology is that of Figure 2. The inferred logic functions use binary values. For example, f(nodeA, nodeB) = nodeA AND nodeB, where nodeA and nodeB have binary (ON or OFF) values. BooleaBayes produces probabilistic functions indicating the probability that the target node will be ON or OFF, depending on the ON/OFF status of its regulators, where a value 0 indicates 100% confidence the target node is OFF, a value 1 indicates 100% confidence the target node is ON, and a value 0.5 indicates equal chance of being ON or OFF. See Figure 3 for an example showing how BooleaBayes finds these values.

Unlike previous work with BooleaBayes, which focused exclusively on transcription factors, the AML network also includes post-translational regulation. For nodes with post-translational regulation, we distinguish between the transcription of the gene, and the activation of the protein product. Protein activation is assumed to follow inhibitory dominant Boolean rules, which means that at least one activator is required, but any inhibitor is enough to prevent activation. Full details of how this was implemented into BooleaBayes are available in Section 2.6.

With a deterministic Boolean model, one may search for its attractors, which represent long-term stable behaviors of the system. Once the system reaches an attractor, it can no longer escape it without an external intervention. As BooleaBayes is a probabilistic system, it has no inescapable attractors. Nevertheless, there are states which the system is more likely to enter than to leave, termed pseudo-attractors.

**Figure 4:**
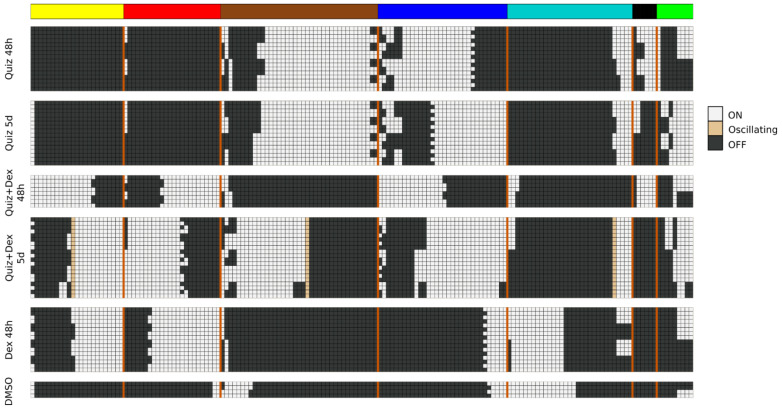
Pseudo-attractors corresponding to DMSO and drug-treated AML, which are also the attractors of the deterministic approximation of the BooleaBayes network. The values of the network’s 65 source nodes are fixed to match one of the six treatment conditions, including DMSO, and attractors of the reduced systems are shown above. Each row is one attractor, and columns are nodes of the network. Columns are grouped and colored based on the module the node belongs in. The vertical orange lines delimit the nodes of each module.

We asked whether the AML network has pseudo-attractors corresponding to the drug-treatment conditions. To find these, we approximated the probabilistic BooleaBayes regulatory functions by finding their closest-matching deterministic Boolean functions (File S4). The network has 65 source nodes (nodes without regulators), which can be 0 or 1 with no constraints, indicating that there will be at least 2^65^ ~= 10^19^ possible attractors (at least one per source node configuration), and even more pseudo-attractors. Not all of these pseudo-attractors necessarily correspond to true attractors of the system, but may instead reflect uncertainty of BooleaBayes functions far from the observed data. To specifically find pseudo-attractors associated with the drug-response, we plugged into the source nodes their respective observed values in each of the drug conditions, and propagated those substitutions to find simplified systems for each drug state. Attractors of these simplified systems were found using the StableMotif [22] Python package (Figure 4). These attractors of the simplified deterministic system correspond to pseudo-attractors of the probabilistic BooleaBayes functions. Pseudo-attractors for the 5-day Quiz+Dex treatment have three oscillating nodes: ABL1_active, CBL_active, and INSR_active, driven by a negative feedback loop between CBL_active and INSR_active. All other pseudo-attractors are steady states. Many modules have clear consensus of the activity of their genes, in which almost all of them are ON or almost all are OFF. For example, almost all yellow nodes and almost all red nodes are OFF in pseudo-attractors corresponding to quizartinib treatment (either for two days or for five days). This agrees with the low module eigengene expression (pink color) on Figure 1B. Other modules are more split. For example, nearly half of the brown and green module nodes are ON and nearly half are OFF in pseudo-attractors corresponding to combination treatment for five days. These also agree with the module eigengene expressions (white color). Overall, there is a good agreement between all modules’ average activation in pseudo-attractors and their eigengene expression shown in Figure 1B.

#### 3.3.3. Identification of Intervention Targets That Disrupt AML Drug Resistance Modules

With the dynamic model of AML drug response, there are many possible questions one could pursue. We focus on identifying interventions that we hypothesize may be able to reprogram DTP cells into drug susceptible states. Specifically, we previously showed that dexamethasone increases cell death of quizartinib-induced DTPs in FLT3-ITD AML [4]. However, there remain gene expression modules that dexamethasone does not reverse ( Figure 1 and Figure 4), including some that are natural markers of DTPs. To identify additional targets that may be able to improve combination quizartinib and dexamethasone treatment, we thus focus on the pseudo-attractors corresponding to combination treatment with Quiz + Dex for 5 days, compared to the attractors in DMSO. As discussed above, several differentially expressed modules are enriched in biological functions that may be responsible for mediating drug resistance. Of greatest interest, the blue module is enriched in detoxification and drug export, the green module is enriched in regulated cell death and apoptosis, and the turquoise module is enriched in cell cycle progression. We hypothesize that downregulating the blue module may prevent the emergence of resistance mechanisms. Activating the turquoise module may enhance proliferation, preventing cells from entering the DTP state. Activating the green module may enhance apoptosis. It is also of note that the yellow and red modules are more highly expressed following Quiz + Dex treatment than DMSO. We hypothesize that reverting these modules to the DMSO state may improve therapy response. Collectively, these changes may extend the efficacy of combination Quiz + Dex treatment.

**Figure 5:**
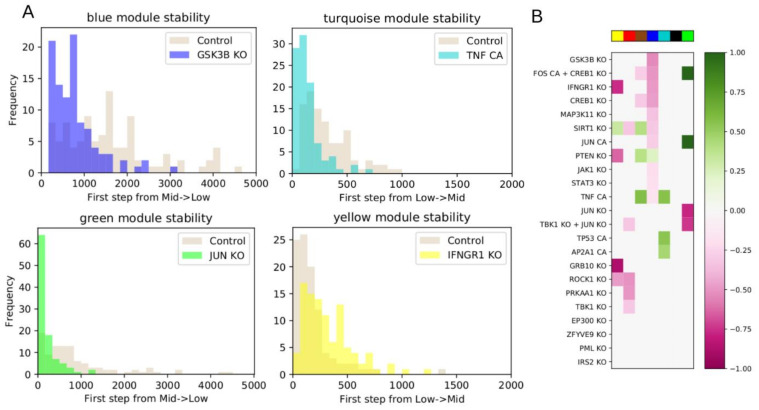
Targeted interventions of driver nodes cause up- or down-regulation of gene modules. (**A**) Distribution of the number of steps required for the module to switch between low and intermediate activation. The distributions in beige show the dynamics of the system with no manipulations, while the colored distributions show the manipulated systems. For modules transitioning from low to intermediate activation, an intervention shifting the distribution to a longer time to switch (rightward shift) maintains the module in the downregulated state, while a leftward shift upregulates the module. For modules transitioning from intermediate to low, a rightward shift indicates the module is maintained in an upregulated state, while a leftward shift indicates downregulation. (**B**) Heatmap showing the up- or down-regulation of statistically significant interventions (two-sided Mann–Whitney U Test, FDR-adjusted *p*-value < 0.05) compared to control for each module. Only significant interventions are colored. Colors are scaled so that a value of +/−1 indicates a 100% relative shift in mean transition time compared to control.

To this end, we picked control objectives of downregulating the blue, red, and yellow modules, and upregulating the turquoise or green modules. Upregulation of the green module was chosen due to its enrichment in apoptosis, even though this would push the green module further away from the DMSO state. We first quantified the stability of the gene expression modules near the Quiz + Dex 5-day pseudo-attractors. To accomplish this, we simulated 100 random walks of 5000 steps (see Methods Section 2.10) from an initial state determined by the average of the Quiz + Dex 5-day pseudo-attractors. For each step along the walk, we quantified the fraction of nodes from each module that are ON to get an overall module activation score. We characterized modules with fewer than 1/4 active nodes to be in a low state, between 1/4 and 3/4 to be in an intermediate state, and above 3/4 to be in a high state. These ranges were chosen to ensure each module began sufficiently far from the boundary. With these definitions, all modules began in either the low or intermediate states. We then quantified how long it took for each random-walk simulation to cross from low to intermediate activation, or from intermediate to low activation, for the first time (we only rarely observed a cross from intermediate to high activation, so this transition was excluded). The distributions of the crossing times in Figure 5A capture the baseline stability of each module.

We then asked which interventions shift the module distributions to lower or higher numbers of steps. If a module starts in the intermediate state and transitions into the low state, then interventions shifting the distribution to a higher number of steps stabilize the more active state of the module. Conversely, interventions shifting the distribution to a lower number of steps downregulate the module. The opposite interpretation holds for modules starting in the low state and transitioning to the intermediate state: a shift to a lower number of steps indicates upregulating the module, while a shift to a higher number of steps indicates maintaining the module in the low state.

Nodes targeted by in silico intervention were fixed as either ON or OFF, and not allowed to update during the simulation. We prioritized nodes to target by (1) analysis of the regulatory paths in the network (Figure 2 and Figure S10), and (2) calculation of an influence index for each possible node interventions and each module (see Methods Section 2.9 for details). Briefly, the influence index considers the most likely effect an intervention of a given node will have on the nodes it targets. A positive influence index indicates that those effects are likely to align with our control objectives, while a negative influence index indicates that those effects are likely to contradict our objectives. Influence indices for each intervention are given in File S5.

We tested the interventions shown in Figure 5 by simulation. For each intervention, we quantified how the distribution of steps required to cross the low-intermediate threshold shifts relative to the baseline control. We determined significant upregulating or downregulating shifts using a two-sided Mann–Whitney U test (see Methods Section 2.10). The most significant regulators for each module are shown in Figure 5B.

We predict several interventions that may lead to the downregulation of the blue module, which is enriched in genes related to drug resistance. The most significant are knockout of GSK3B, IFNGR1, CREB1, SIRT1, or MAP3K11. Investigating these further, GSK3B inhibition has previously been proposed as a differentiation-inducing therapy for AML [28,29]. Nevertheless, it has also been found in a CRISPR screen that GSK3B KO leads to the reactivation of FGF/Ras/ERK and Wnt signaling that can confer resistance to quizartinib monotherapy in *FLT3*-ITD AML [30]. CREB1 overexpression has been associated with poor outcome in AML patients [31], and SIRT1 activation has been previously associated with drug resistance of *FLT3*-ITD AML stem cells [32].

For the green module, which is enriched in apoptosis regulation, we find JUN constitutive activation leads to activation of the green module, while JUN knockout inhibits it. Previous work has found that JUN KO increased apoptosis in AML cells [33]. JUN is a master regulator of apoptosis, but also involved in AML cell survival via inflammatory pathways, indicating it may have dual roles. Expanding the network to include relevant downstream JUN activity may better elucidate how these competing effects may be activated or controlled.

For the turquoise module, which is enriched in cell cycle progression, we found activation of TNF, TP53, or AP2A1 support upregulation of the turquoise module. TNF-alpha is highly upregulated in AML patients, and has been shown to induce proliferation of leukemic blasts [34,35]

We additionally tested combination interventions to simultaneously control multiple gene modules. The combined knockout of TBK1 and JUN and found that it leads to downregulation of both the red and yellow modules. Simultaneous constitutive activation of FOS with knockout of CREB1 led to downregulation of the brown and blue modules, and stabilization of the green module.

Focusing on modules other than blue, green, and turquoise, we predict that GRB10 KO strongly downregulates the yellow module, which is upregulated by both quizartinib and dexamethasone treatment, and GRB10 overexpression has previously been associated with aggressive phenotypes in *FLT3*-ITD AML [36]. ROCK1 KO downregulates both the yellow and red modules, and ROCK inhibition has been shown to inhibit cell growth in *FLT3* mutant AML patient-derived blasts [37]. We predict that TBK1 KO downregulates the red module; it has previously been suggested as a therapeutic target in AML due to its activation of MYC-dependent survival pathways [38].

Four interventions had relatively high influence indices but did not lead to a statistically significant shift: KO of EP300, ZFYVE9, PML, or IRS2. Of these, ZFYVE9 and IRS2 KO have a large effect on their direct targets, but that effect clearly will not propagate through the network. For instance, ZFYVE9 is a necessary regulator for two blue module genes: SMAD2 and SMAD3, which BooleaBayes did not detect significant regulatory functions for in this network, and thus they became sink nodes. IRS2 is necessary for PIK3CA to activate, and PIK3CA_A only regulates a single target node, PTK2B_T. PTK2B_T is also regulated by SRC, and SRC has a much stronger regulatory influence than PIK3CA (File S3). The remaining two interventions, EP300 and PML KO, have multiple downstream paths, but nevertheless our full simulations of the network did not detect upregulating or downregulating influences of these interventions on any module.

The concordance of our identified interventions with previous work from the literature supports the validity of our findings. Furthermore, we make several additional novel predictions. For example, we predict the blue module can be downregulated by the KO of IFNGR1 or MAP3K11. The turquoise module can be upregulated by CA of AP2A1 or TP53. The validation of these novel predictions is needed as a next step in establishing the predictive value of this model and is the subject of future work in our labs. Collectively, we anticipate that these interventions would synergize with combination quizartinib and dexamethasone treatment in patients with *FLT3*-ITD AML.

## 4. Discussion

Here we constructed a dynamic model of a gene regulatory network relevant to *FLT3*-mutant acute myeloid leukemia. The model integrates multiple types of information: RNAseq data consisting of MV4-11 cells exposed to drug treatment and several databases of signal transduction and gene regulation. Model development included multiple state of the art analysis methodologies: weighted gene co-expression analysis, ontology analysis, inference of regulatory relationships using BooleaBayes, attractor analysis, and control theory. We also developed new capabilities for BooleaBayes, and new confidence scores to prioritize interactions to be included in the network and new influence scores to prioritize interventions. Overall, this work illustrates the challenges and capabilities of computational systems biology analysis in cancer research and the potential for this type of analysis to advance personalized medicine.

The model attractors recapitulate the activation of the modules (compare Figure 4 to Figure 1B), and the most significant predicted model interventions match well with literature reports on drivers of proliferation, survival, and drug resistance (Figure 5). Collectively, these results strongly support the model’s validity. Nevertheless, there are several possible avenues for further model improvement. This model was derived from data in MV4-11 cells treated with quizartinib and dexamethasone. We previously showed that the gene expression profile of MV4-11 cells was predictive of sensitivity of multiple *FLT3*-ITD cell lines and patient cells to treatment with quizartinib and dexamethasone [4]. Nevertheless, including data collected from other cell lines, or cells treated with other drugs, such as other *FLT3* inhibitors or glucocorticoids may reveal alternative pathways and processes involved in mediating drug resistance. Finding common resistance mechanisms, as well as system-specific resistance mechanisms, may lead to a more generalizable model. Furthermore, during network construction we removed sink nodes to focus on nodes that contribute feedback into the network dynamics. Nevertheless, those sink nodes may be valuable phenotypic markers, or could be regulators of other nodes we may include in the future. Additionally, the large number of source nodes (65) should eventually be decreased. Many of these became source nodes because BooleaBayes was not able to determine a significant role for their regulators, and so those edges were removed. Additional expression datasets, or literature knowledge, may elucidate functional forms of those interactions. Additionally, more nodes may be added by including more AML-specific literature knowledge (e.g., MCL as downstream target of GSK3B, downstream targets of JUN to further elucidate the dual effect of its inhibition on apoptosis and inflammation).

In Figure 5 we showed four interventions that had high influence indices, but this did not translate into significant up- or down-regulation of any modules. In at least two cases, we determined that these interventions led to sink nodes, or nodes with weak influence, explaining why the influence index was not predictive of overall impact. To address this, the influence index of a node may be extended to consider the influence index of its downstream targets. Further, nodes can have conflicting downstream effects, and resolving these may improve the predictive value of influence index.

The dynamic model may eventually be used to answer other fundamental questions, such as how does drug treatments lead to the resistant state. To this end, the network could be extended by integrating known drug targets, though in practice drugs often have multiple off-target effects. One possible way to overcome this would be to prioritize adding drug targets that can induce the changes between the status of the source nodes in the untreated and drug-treated conditions. Future work is focused on expanding and improving the network model by incorporating information about drug targets, additional cell lines, and additional drug perturbation datasets. We are also working to validate the model’s novel predictions, such as combining Quiz+Dex treatment with KO of IFNG1 or MAP3K11, or CA of AP2A1.

Finally, we anticipate that data-driven predictive modeling, as demonstrated in this work, may eventually help accelerate patient-specific precision treatments. The dynamics of the AML model emerged from the expression data we used to train it, thus incorporating patient-specific data may help reveal patient-specific drug resistance pathways or targets.

## Data Availability

No new data were created or analyzed in this study. Data sharing is not applicable to this article.

## Supplementary Materials

The following are available online at https://www.mdpi.com/2075-4426/11/3/193/s1, Figure S1: WGCNA module identification, Figure S2: Black module enrichment, Figure S3: Blue module enrichment, Figure S4: Brown module enrichment, Figure S5: Green module enrichment, Figure S6: Red module enrichment, Figure S7: Turquoise module enrichment, Figure S8: Yellow module enrichment, Figure S9: Network construction, Figure S10: Full network, File S1.csv: WGCNA modules, File S2.xlsx: MBCO enrichment, File S3.txt: BooleaBayes fit probabilistic update functions, File S4.txt: Deterministic Boolean approximation, File S5.csv: Influence indices.
