# Supplementary material for: Data-Driven Math Model of FLT3-ITD Acute Myeloid Leukemia Reveals Potential Therapeutic Targets
Source: J Pers Med. 2021 Mar 11;11(3):193. doi: 10.3390/jpm11030193 (PMC7998618; doi:10.3390/jpm11030193)
Supplement: Supplementary file 1 [file jpm-11-00193-s001.zip › Supplemental Files/SupplementalFileCaptions.docx]

Data-driven math model of FLT3-ITD acute myeloid leukemia reveals potential therapeutic targets

Supplemental Materials

**Figure S1.** WGCNA module identification. Identification of gene co-expression modules in control and drug-treated MV4-11 cell lines. Left panel shows WGCNA scale independence. We chose a soft threshold of 10 to achieve R^2 > 0.9. Right panel shows gene clustering and module assignment using the WGCNA dynamic tree cut algorithm.

**Figure S2.** Black module enrichment. MBCO enrichment analysis on genes from the black module. Colors indicate MBCO ontology level (*i.e.*, 1-3), and shade indicates whether the ontology was enriched on its own, or only through interactions with other ontologies. Full enrichment results are in File S2.

**Figure S3**. Blue module enrichment. MBCO enrichment analysis on genes from the blue module. See Figure S2 caption for more details.

**Figure S4.** Brown module enrichment. MBCO enrichment analysis on genes from the brown module. See Figure S2 caption for more details.

**Figure S5.** Green module enrichment. MBCO enrichment analysis on genes from the green module. See Figure S2 caption for more details.

**Figure S6.** Red module enrichment. MBCO enrichment analysis on genes from the red module. See Figure S2 caption for more details.

**Figure S7.** Turquoise module enrichment. MBCO enrichment analysis on genes from the turquoise module. See Figure S2 caption for more details.

**Figure S8.** Yellow module enrichment. MBCO enrichment analysis on genes from the yellow module. See Figure S2 caption for more details.

**Figure S9**. Network construction. (A) Workflow showing construction of AML drug response network from resource networks. (B) Example showing a node, C, that exists on a path between nodes A and B. To connect disconnected segments of the merged yellow, brown, green, black, and red subgraphs, we included edges like this that had at least one node from those modules, and no more than 3 additional nodes. Nodes X, Y, and Z in this example may be from the blue or turquoise modules, or may not have been assigned to a module by WGCNA.

**Figure S10.** Full network. Full network used for BooleaBayes training, with some genes separated into gene_T (representing the transcript), and gene_A (representing activated form of the protein encoded by the gene).

**File S1.** WGCNA gene-module assignments.

**File S2.** MBCO dynamic enrichment analysis results for each WGCNA module.

**File S3.** BooleaBayes inferred probabilistic update functions. Each line is in the format “Target|Regulator1,Regulator2,…,RegulatorN|a,b,c,d,e,…,z”. Target is the gene being regulated, and it is regulated by all the listed regulators. The numbers indicate the probability for the target to turn ON in different conditions. If a node has 3 regulators (A,B,C), then there will be 8 probabilities listed, corresponding to the 8 regulator input conditions. From left to right, these conditions will be (0,0,0), (0,0,1), (0,1,0), (0,1,1), (1,0,0), (1,0,1), (1,1,0), (1,1,1). This same pattern holds for nodes with more or fewer regulators as well.

**File S4.** Deterministic Boolean approximation. Each of the BooleaBayes inferred probabilistic functions is transformed to the closest deterministic function. These are written in BooleanNet format.

**File S5.** Influence indices calculated for each node, intervention, and target module. Positive values indicate the intervention is likely to lead to effects aligning with the goal for a module, while negative values indicate the intervention is likely to counteract the goal for a module.
