# Supplementary figures and images for: Data-Driven Math Model of FLT3-ITD Acute Myeloid Leukemia Reveals Potential Therapeutic Targets
Source: J Pers Med. 2021 Mar 11;11(3):193. doi: 10.3390/jpm11030193 (PMC7998618; doi:10.3390/jpm11030193)

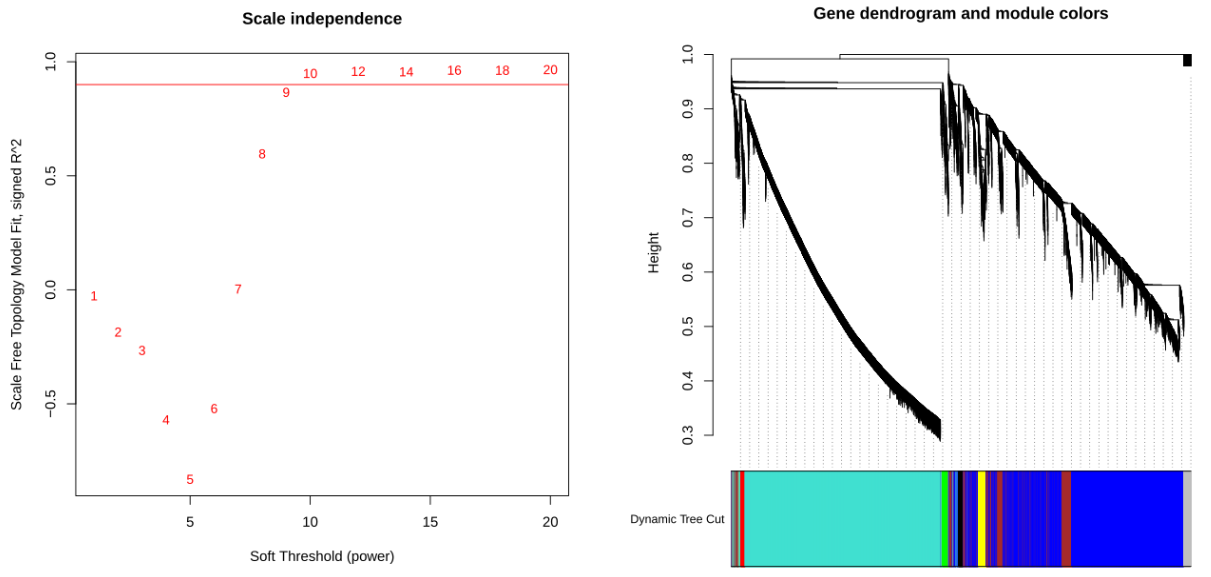

Supplement: Supplementary file 1 [file jpm-11-00193-s001.zip › Supplemental Files/Figure S1.png]

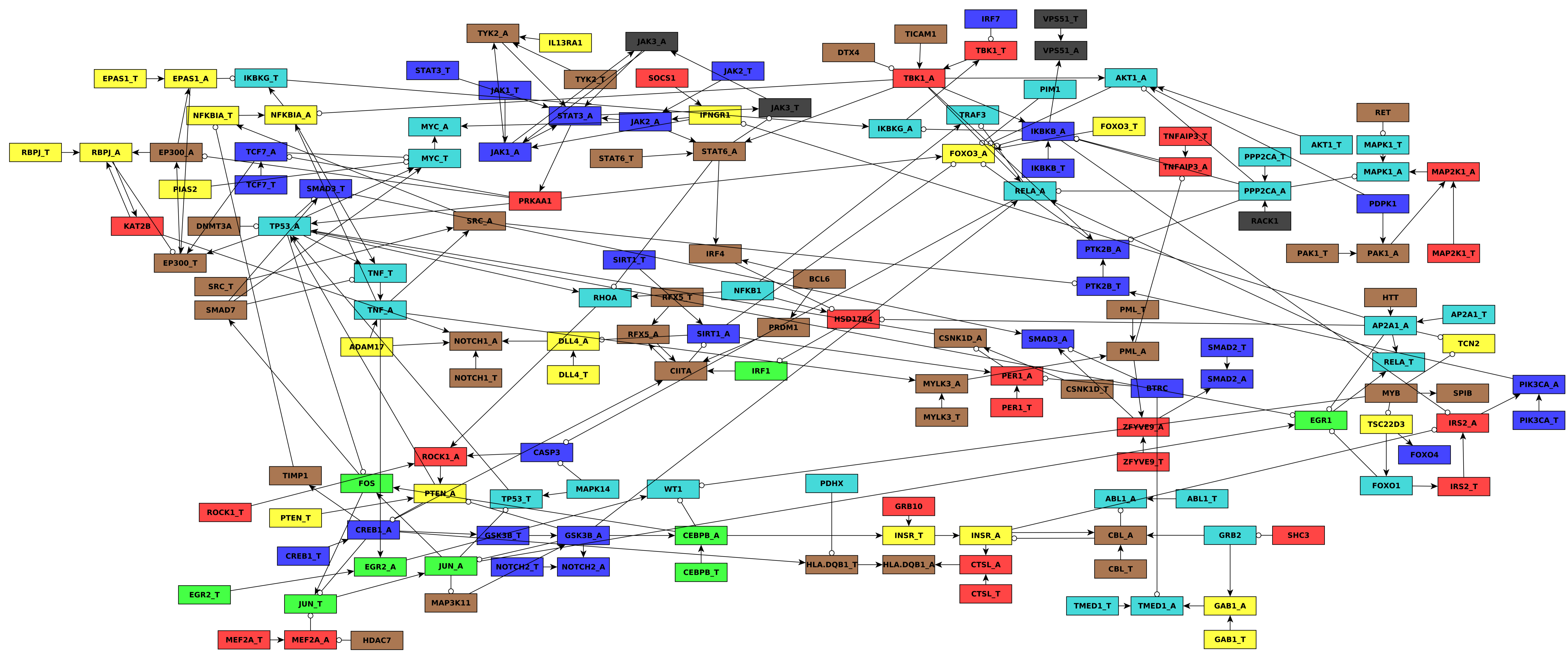

Supplement: Supplementary file 1 [file jpm-11-00193-s001.zip › Supplemental Files/FIgure S10.pdf]

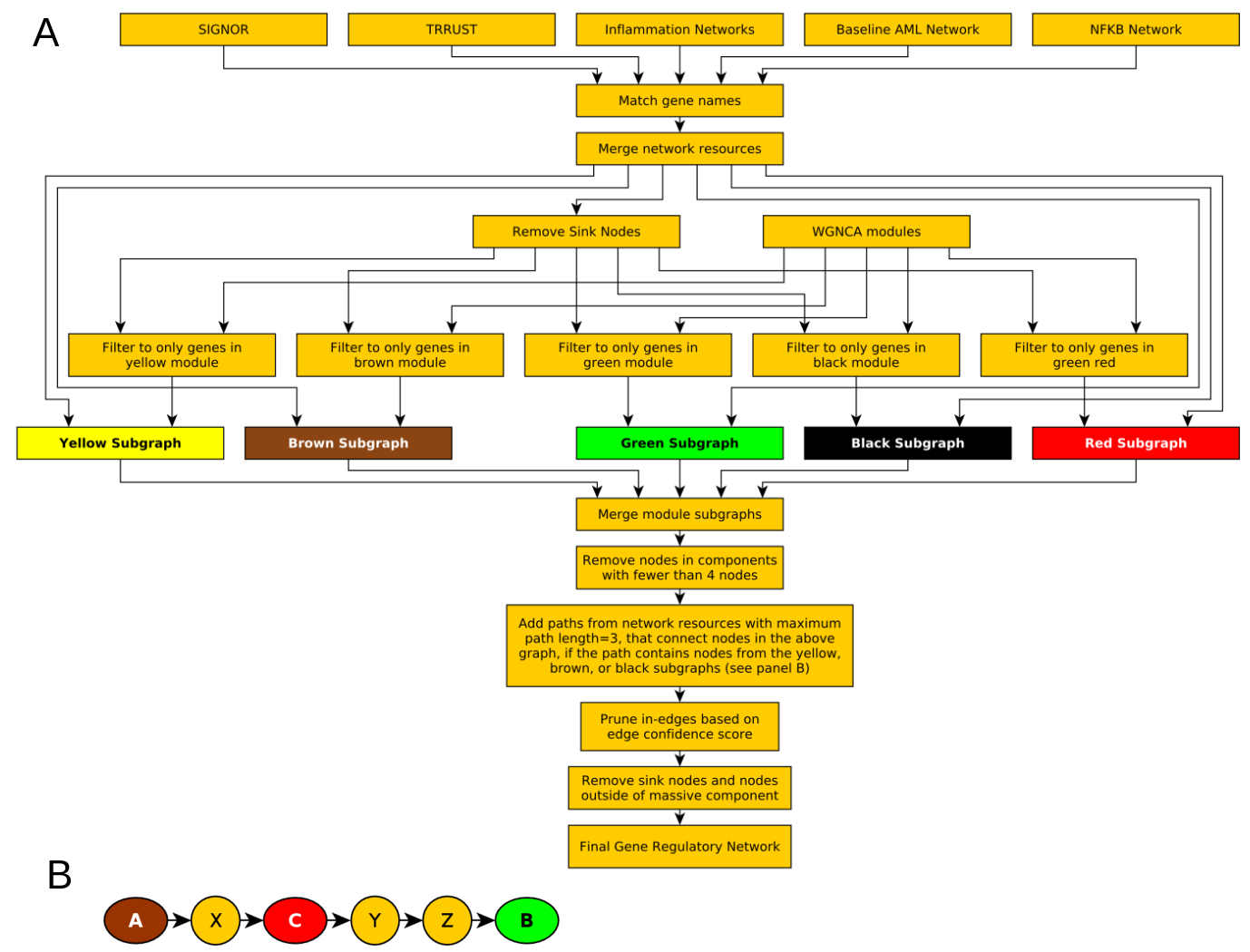

Supplement: Supplementary file 1 [file jpm-11-00193-s001.zip › Supplemental Files/FIgure S9.png]
